# Analytical Models for Grain Size Determination of Metallic Coatings and Machined Surface Layers Using the Four-Point Probe Method

**DOI:** 10.3390/ma16176000

**Published:** 2023-08-31

**Authors:** Thomas Mehner, Thomas Lampke

**Affiliations:** Materials and Surface Engineering, Institute of Materials Science and Engineering, Chemnitz University of Technology, Erfenschlager Str. 73, D-09125 Chemnitz, Germany; thomas.lampke@mb.tu-chemnitz.de

**Keywords:** electrical resistance, electrical resistivity, electrical conductivity, grain size, analytical model, four-point probe method, surface layer

## Abstract

The grain size of a metallic coating or the surface layer after the machining of metallic parts strongly impacts corrosion and wear properties along with fatigue behavior. By measuring the combined electrical resistance of this layer and the substrate using the four-point probe method, the grain size of the layer can be determined. For different grain shapes, models are derived based on an analytical approach. The parameters in the models can be determined by appropriate calibration measurements. As a result, the grain sizes can be determined quickly with a non-destructive method, which can be applied to ensure consistent coating or machining results as part of quality control routines in industrial processes.

## 1. Introduction

In recent years, surface integrity has become a focus of attention. Surface integrity can be enhanced by surface conditioning during machining, if proper methods are applied [1]. By machining, a surface layer is created, which differs from the bulk material underneath. While the effect of this layer on the material properties is significant, quick methods for non-destructive investigations are barely available. They include, e.g., Barkhausen noise analysis, eddy current testing, and electrical measurements using the four-point probe method, e.g., [2,3,4,5].

One important property impacted by the surface layer is the fatigue strength, which depends on the crack propagation and thus on the grain size. Electrical measurements are promising regarding the correlation with grain size. Mayadas and Shatzkes proposed a model for electrical resistivity, which describes the impacts of the grain boundary, surface, and interface scattering on the resistivity [6]. These processes are most important in nm-thick layers. Along with additional models by Tellier [7] and Bedda et al. [8], this leads to the conclusion that above a layer thickness of about 200 nm, the resistivity is almost constant. This behavior could be experimentally validated in thin films, where the electrical resistivity depended on the layer thickness (i.e., the grain size) of Cu [9,10,11], Ti [12], and W [13] amongst others. In nanocrystalline Gd, the electrical resistivity was shown to double in samples with grain sizes of 100 nm compared to 1 µm [14]. Based on these studies, the electrical resistivity of machined surfaces (with typical grain sizes >> 1 µm) is expected to be independent of the grain size.

However, in Mg wires, defect-rich grains showed a higher resistivity after the wire-drawing process compared to the values after an additional heat treatment [3]. This was observed most notably with short annealing times (<20 s) accompanied by grain sizes below 20 µm. A similar effect was observed by Yoo et al. after the annealing of Co-W nanowires [15]. Santos et al. [16] as well as Metan and Eigenfeld [17] found a lower conductivity in fine-grained areas compared to non-refined ones. Barghout showed a reduction in the electrical conductivity in Al-Fe wires when the grain size was below 10 µm [18]. In Cu-Fe alloys, an increased conductivity was found after heat treatment, i.e., a grain size increase [19]. In most of those studies, the measured decrease was attributed to grain boundaries (GBs). Bishara et al. studied the effect of GBs on the electrical resistivity in great detail [20]. They found a significant increase in the resistivity of GBs, which depends, among other things, on the curvature of the GB and the coincidence site lattice type of the GB.

In a recent publication by the authors, an analytical model was published, which allows for the determination of grain sizes in homogeneous materials by measuring their electrical resistance using the four-point probe method [21]. In this model, rows of grains in different shapes (cylindrically shaped; spherically shaped) are connected in parallel, which allows for the calculation of the electrical resistance and, inversely, the grain size based on the measured electrical resistance. However, it is not applicable to non-homogeneous samples and it does not include GBs. In addition, the model assumes “empty volumes” between the rows of grains, which is not observed in real samples. Nonetheless, the agreement between the model and measurements of homogeneous, rolled samples was shown to be very good.

Based on this model, extended models are developed within the present study, which can be applied to metallic materials and which account for the mentioned restrictions. The models are not suitable for semiconducting and electrical insulating materials. They explicitly model GBs as a material volume of increased electrical resistivity, fill the volumes between the rows of grains with rows of a second type of grain, and add a dependency of the electrical resistance on the distance of the row to the four-point probe tip. Therefore, it is possible to model systems that consist of a (machined) surface layer on metallic bulk material or a metallic coating on a metallic substrate with grain sizes > 1 µm. In the following, the modeling is presented in detail for three grain shapes/orientations. The detailed description allows for an easy adaptation to other grain shapes (e.g., ellipsoids).

## 2. Materials and Methods

**Figure 1:**
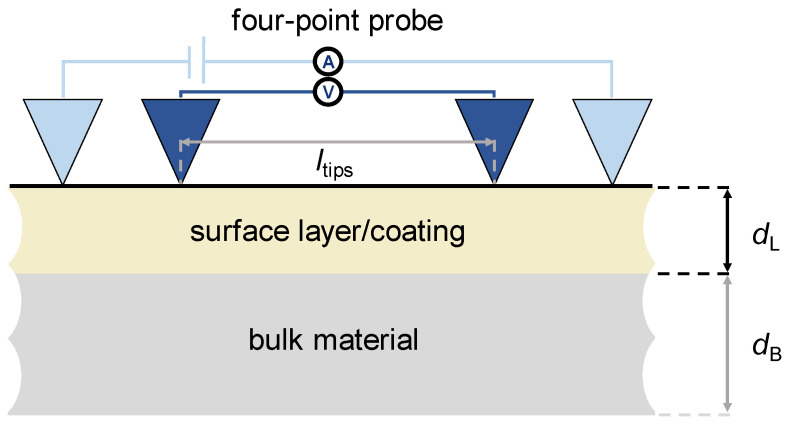
Schematic setup for the measurement of the electrical resistance for samples with a surface layer/coating (thickness *d*_L_) on top of the bulk material (thickness *d*_B_) using the four-point probe method (*l*_tips_—distance between the inner tips).

Based on the above-mentioned previous model for homogeneous grain sizes within the entire cross-section [21], the modeling is extended to account for local differences of the material, phases, and grain sizes. Figure 1 shows the measurement setup schematically, which is used in the modeling.

**Figure 2:**
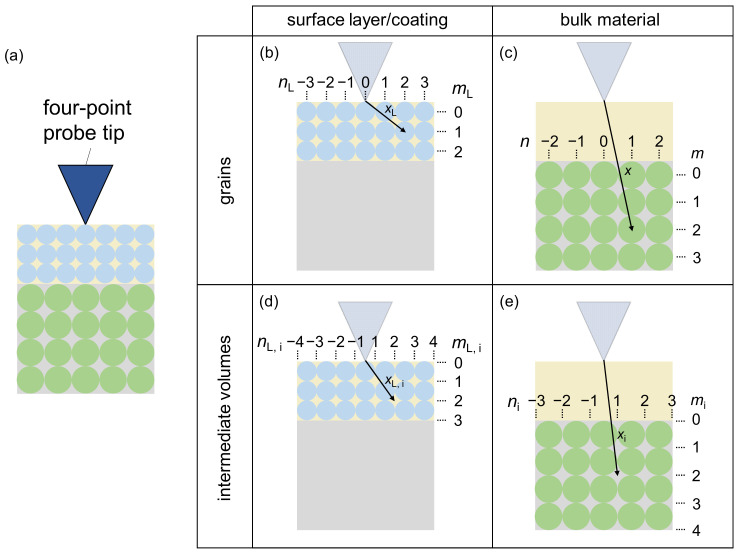
Indexation for the modeling (**a**) schematic cross-section view of the material perpendicular to the view in Figure 1 (surface layer/coating—tan, grains—blue; bulk material—gray, grains—green); (**b**) grains in the surface layer/coating; (**c**) grains in the bulk material; (**d**) intermediate volumes in the surface layer/coating; (**e**) intermediate volumes in the bulk material. The arrows indicate examples of the distance between the tip and one row of grains or intermediate volumes.

The model generally assumes that the material consists of identical grains (size, shape, resistivity), which are themselves homogeneous and aligned in rows. These rows fill the cross-section of the surface layer/coating and the bulk material as shown in Figure 2a. In between the grain rows, the model assumes another type of grain, which fills the intermediate volumes in order to avoid empty spaces within the material (Figure 2d,e). It also assumes that the current cannot pass through high-angle grain boundaries, which are aligned parallel to the direction of the measuring current (outer tips in Figure 1). The individual rows of grains and intermediate volumes are indexed as shown in Figure 2b–e for modeling purposes (*m* ${\in N}_{0}$, *n* ∈Z). The indexes are as follows: L—layer; i—intermediate volumes; max—highest value of the index.

## 3. Results

**Figure 3:**
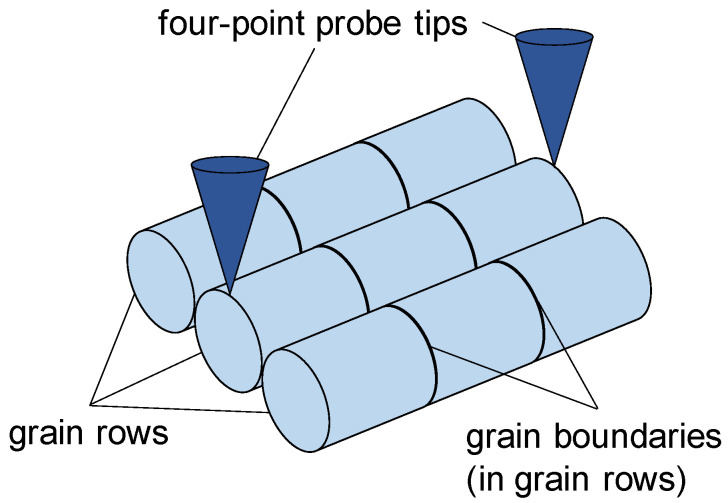
Schematic view of the assumed model geometry with the example of three grain rows of cylindrically shaped grains (light blue) with grain boundaries (black). The electrical resistance of each row is impacted by the distance of the grain’s center of the base plane to the four-point probe’s tip (dark blue).

In this section, the modeling is described in detail. As in the previous model [21], the electrical resistance is calculated as the electrical resistance of parallel connected rows, whereby the individual rows are grains connected in series. The rows of grains do not interact with each other. An example of this model geometry is shown in Figure 3 for three rows, each consisting of three cylindrically shaped grains. Integer numbers of grains and grain boundaries within *l*_tips_, *d*_L_, and *d*_B_ are assumed. This is indicated by […]_int_ in the formulas, i.e., rounded down to the nearest integer value. The grain boundaries (index gb) within each row are assumed to have an electrical (volume) resistivity *ρ*_gb_, which is increased by a factor of *f* compared to that of the grains *ρ*, i.e.,

(1) $$\rho_{gb}=f\cdot \rho \;\;(\mathrm{with}\;f\;\geq \;1).$$

The grain boundary between two grains has a thickness of *l*^gb^ (with *l*^gb^ << *l*_tips_). Thus, *l*_tips_ consists of a fraction of grain boundaries and a fraction of grains. This is shown in detail in the modeling sections for different grain shapes.

Furthermore, the model presented here should be able to differentiate the surface layer/coating from the bulk material. Thus, the distance of each row of grains to the tip of the four-point probe directly under the tip is considered (*x*, *x*_L_, *x*_i_, *x*_L,i_ in Figure 2). It is assumed that rows further away from the tip have a lower impact on the measured voltage drop at the four-point probe tip, i.e., that their measured electrical resistance increases with increasing distance. The factor *P* of this resistance increase is assumed to be:

(2) $$P={\left(\frac{X}{C}\right)}^{\gamma }$$

with the distance *X* ∈ {*x*, *x*_L_, *x*_i_, *x*_L,i_}, a material-dependent, geometry-independent, resistance-increasing factor *C* ∈ {*c*, *c*_L_, *c*_i_, *c*_L,i_}, and a material-dependent, geometry-independent resistance-increasing power *γ*. Then, all grains and grain boundaries in one row of grains are connected in series to calculate the electrical resistance of the row (*R*_m,n_), which is subsequently modified by the factor *P*_m,n_ to account for the distance of the row to the tip.

### 3.1. Modeling of Different Grain Shapes

At first, models for different grain shapes are presented, namely cylindrically shaped grains in two different orientations and spherically shaped grains. They are derived independently of their position (surface layer/coating or bulk material). These models can also be used for homogeneous layers. Later on, they are combined with the factor *P* to allow for their use in systems with a surface layer/coating on a bulk material/substrate. 

#### 3.1.1. Resistance Measurement Parallel to the Cylinder Axis (CA) of Cylindrically Shaped Grains

Cylindrically shaped grains (index c) are characterized by two parameters: *r*_c_—radius of the cylinder’s base plane and *h*—height of the cylinder. Within *l*_tips_, the number of grain boundaries (*N*_gb,CA_) can be calculated by:

(3) $$N_{gb,CA}={\left[\frac{l_{tips}}{h}\right]}_{int}.$$

Using the well-known general formula for the electrical resistance of, e.g., a wire (*l*—length and *A*—cross-sectional area of the wire):

(4) $$R=\rho \cdot \frac{l}{A},$$

Under the addition of grain boundaries (Equation (1)), the total resistance of one row of cylindrically shaped grains along the cylinder axis becomes:

(5) $$R^{CA}=\frac{\rho }{\pi \cdot r_{c}^{2}}\cdot \left(l_{tips}-{\left[\frac{l_{tips}}{h}\right]}_{int}\cdot l_{CA}^{gb}\right)+\frac{f\cdot \rho }{\pi \cdot r_{c}^{2}}\cdot {\left[\frac{l_{tips}}{h}\right]}_{int}\cdot l_{CA}^{gb}=\frac{\rho }{\pi \cdot r_{c}^{2}}\cdot \left(l_{tips}-{\left[\frac{l_{tips}}{h}\right]}_{int}\cdot l_{CA}^{gb}\cdot \left(1-f\right)\right).$$

**Figure 4:**
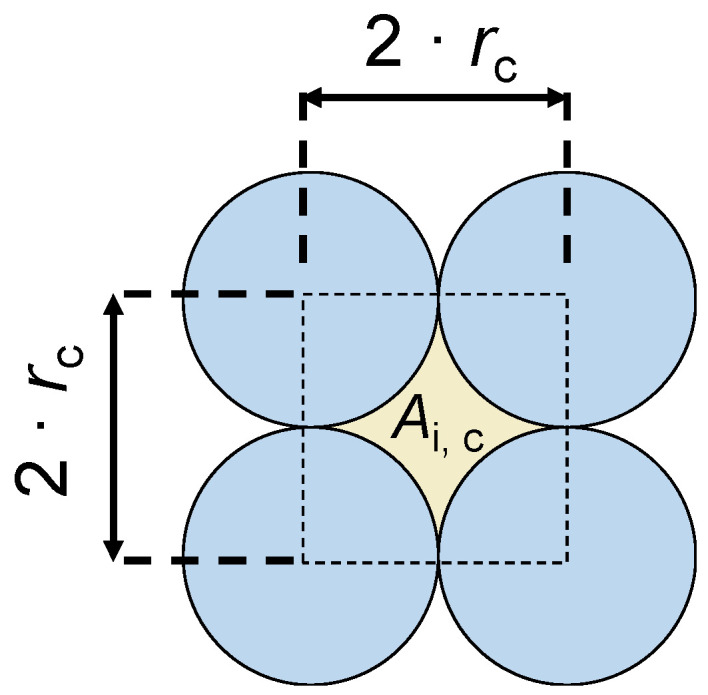
Determination of the area *A*_i,c_ of the intermediate volumes (cylindrically shaped grains).

Equation (5) allows for the calculation of the electrical resistance of one row of grains independent of its *m* and *n* values. In a similar way, the resistance of the intermediate volumes can be derived. However, their shape is more complex. The determination of their area *A*_i,c_ can be performed based on Figure 4. 

The area *A*_i,c_ can then be calculated by:

(6) $$A_{i,c}={\left(2\cdot r_{c}\right)}^{2}-\pi \cdot r_{c}^{2}=\left(4-\pi \right)\cdot r_{c}^{2}.$$

Their height is assumed to be *h*, as well. Thus, the total resistance of one row of intermediate volumes (including grain boundaries) is given by:

(7) $$R_{i}^{CA}=\frac{\rho }{\left(4-\pi \right)\cdot r_{c}^{2}}\cdot \left(l_{tips}-{\left[\frac{l_{tips}}{h}\right]}_{int}\cdot l_{CA}^{gb}\cdot \left(1-f\right)\right).$$

#### 3.1.2. Resistance Measurement Perpendicular to the Cylinder Axis of Cylindrically Shaped Grains

**Figure 5:**
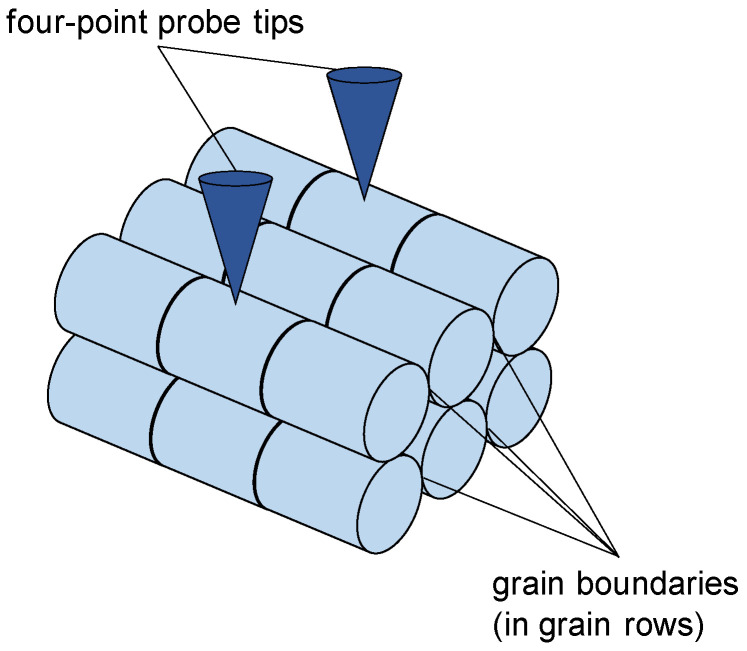
Schematic view of the assumed model geometry with the example of six grain rows of cylindrically shaped grains (light blue) with grain boundaries (black). The resistance is measured perpendicular to the cylinder axis of cylindrically shaped grains. The four-point probe tips touch the grains in the center.

Figure 5 shows the geometry in the case of a resistance measurement perpendicular (index perp) to the CA of cylindrically shaped grains. In this geometry, no connected rows of intermediate volumes are expected to be present in the direction of the measurement. Thus, intermediate volumes are neglected here.

**Figure 6:**
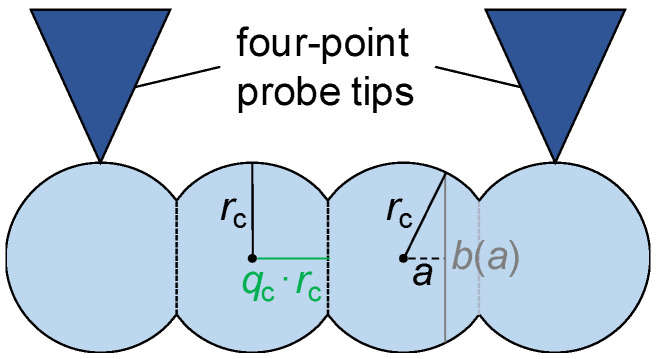
Schematic view of the flattening of the cylindrically shaped grains (four grains pictured) in the horizontal direction (flattening factor 0 < *q*_c_ < 1) and geometric considerations for the integration needed to determine the electrical resistance of one flattened grain (integration parameter *a* along the reduced radius and corresponding chord *b*(*a*)). Model-relevant grain boundaries between the grains are indicated as dashed lines.

A resistance of the grain boundary can only be determined if the contact between two grains is not simply a line. Thus, a flattening of the grains has to be assumed in order to form a rectangular contact area, i.e., the radius *r*_c_ is reduced in one direction (Figure 6). 

As described in [21], an integration is needed to determine the electrical resistance of one flattened grain *R*_g,perp_: (index g—grain; the factor of 2 is due to integration starting from *a* = 0 instead of *a* = −*q*_c_ · *r*_c_):

(8) $$\begin{array}{c} R_{g,perp}=2\cdot \int_{a=0}^{q_{c}\cdot r_{c}}\frac{\rho }{h\cdot b\left(a\right)}da=\frac{\rho }{h}\cdot \int_{a=0}^{q_{c}\cdot r_{c}}\frac{da}{\sqrt{r_{c}^{2}-a^{2}}}={\left.\frac{\rho }{h}\cdot \arctan\left(\frac{a}{\sqrt{r_{c}^{2}-a^{2}}}\right)\right|}_{a=0}^{q_{c}\cdot r_{c}}\\ =\frac{\rho }{h}\cdot arctan\left(\frac{q_{c}}{\sqrt{1-{q_{c}}^{2}}}\right)=:\frac{\rho }{h}\cdot B_{c}\left(q_{c}\right)\;\left({\mathrm{with}\;0<q}_{c}<1\right). \end{array}$$

The resistance of one grain boundary can be calculated using Equations (1) and (4):

(9) $$R_{gb,perp}=f\cdot \frac{\rho }{h}\cdot \frac{l_{perp}^{gb}}{2\cdot r_{c}\cdot \sqrt{1-q_{c}^{2}}}.$$

Due to the grain geometry, the number of grain boundaries *N*_gb,perp_ and the number of grains *N*_g,perp_ in each row has to be determined in order to calculate the electrical resistance of the row:

(10) $$N_{gb,perp}={\left[\frac{l_{tips}}{{2\cdot q}_{c}\cdot r_{c}}\right]}_{int},$$

(11) $$N_{g,perp}={\left[\frac{{l_{tips}-\left[\frac{l_{tips}}{2\cdot q_{c}\cdot r_{c}}\right]}_{int}\cdot l_{perp}^{gb}}{2\cdot q_{c}\cdot r_{c}}\right]}_{int}=N_{gb,perp}\left(l_{perp}^{gb}\ll l_{tips}\right).$$

The two integer numbers are equal due to the assumed geometry and as *N*_g,perp_ > 1 (Figure 6). Thus, the total resistance of one row of grains can be calculated by:

(12) $$R^{perp}=N_{g,perp}\cdot {(R}_{g,perp}+R_{gb,perp})={\left[\frac{l_{tips}}{{2\cdot q}_{c}\cdot r_{c}}\right]}_{int}\cdot \frac{\rho }{h}\cdot \left(B_{c}\left(q_{c}\right)+\frac{f\cdot l_{perp}^{gb}}{2\cdot r_{c}\cdot \sqrt{1-{q_{c}}^{2}}}\right).$$

#### 3.1.3. Resistance Measurement of Spherically Shaped Grains

**Figure 7:**
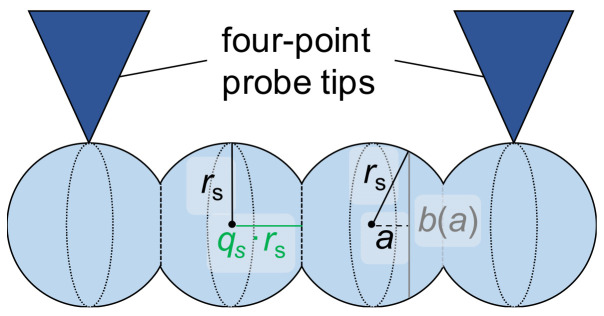
Schematic view of the flattening of spherically shaped grains (four grains pictured) in the horizontal direction (flattening factor *q*_s_ < 1) and geometric considerations for the integration needed to determine the electrical resistance of one flattened grain (integration parameter *a* along the reduced radius and corresponding chord *b*(*a*)). Model-relevant grain boundaries between the grains are indicated as dashed lines.

The resistance of the grain boundary between spherically shaped grains (index s) can only be determined if the contact between two grains is not simply a single point. Similar to the previous section, a flattening of the grains has to be assumed. The radius in one direction is reduced so that a circular contact area is present (Figure 7).

The electrical resistance of one flattened, spherically shaped grain has previously been calculated (based on the geometric consideration in Figure 7) to be [21]:

(13) $$R_{g,s}={\left.\frac{2\cdot \rho }{\pi \cdot r_{s}}\cdot \mathrm{artanh}\left(\frac{a}{r_{s}}\right)\right|}_{a=0}^{q_{s}\cdot r_{s}}=\frac{2\cdot \rho }{\pi \cdot r_{s}}\cdot \mathrm{artanh}\left(q_{s}\right)=∶\frac{2\cdot \rho }{\pi \cdot r_{s}}\cdot B_{s}\left(q_{s}\right)\;\left({\mathrm{with}\;q}_{s}<1\right).$$

Some values of the area hyperbolic tangent (artanh) are given in [21]. The resistance of one grain boundary can be calculated using Equations (1) and (4):

(14) $$R_{gb,s}=f\cdot \rho \cdot \frac{l_{s}^{gb}}{\pi \cdot r_{s}^{2}\cdot \left(1-q_{s}^{2}\right)}.$$

Analogous to Equation (10) and the geometric considerations in Figure 7, the number of grains and grain boundaries are given by:

(15) $$N_{g,s}=N_{gb,s}={\left[\frac{l_{tips}}{{2\cdot q}_{s}\cdot r_{s}}\right]}_{int}.$$

Thus, the total resistance of one row of spherically shaped grains can be calculated by:

(16) $$R^{s}=N_{g,s}\cdot {(R}_{g,s}+R_{gb,s})={\left[\frac{l_{tips}}{{2\cdot q}_{s}\cdot r_{s}}\right]}_{int}\cdot \frac{\rho }{\pi \cdot r_{s}}\cdot \left(2\cdot B_{s}(q_{s})+\frac{{f\cdot l}_{s}^{gb}}{r_{s}\cdot \left(1-q_{s}^{2}\right)}\right).$$

**Figure 8:**
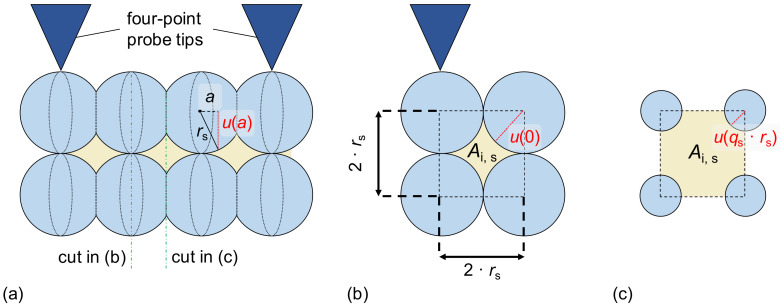
Schematic view of rows of flattened, spherically shaped grains. (**a**) Side view for the determination of *u*(*a*) (*a*—integration parameter), (**b**) cross-sectional view through the radius grains (*a* = 0), and (**c**) cross-sectional view through the contact point of the grains (*a* = *a*_max_ = *q*_s_ · *r*_s_).

The intermediate volumes between spherically shaped grains have a more complex shape compared to cylindrically shaped grains due to a non-constant cross-section. Figure 8 shows the relevant geometry and defines the necessary parameters for the following calculation of the electrical resistance. It is assumed that adjacent grains do not interact, i.e., that the high-angle grain boundary between neighboring rows of grains is sufficiently electrically insulating.

Thus, the electrical resistance *R*_i,g,s_ of one grain of intermediate volume can be calculated by (the factor of 2 is due to integration starting from *a* = 0 instead of *a* = −*q*_s_ · *r*_s_):

(17) $$\begin{array}{c} R_{i,g,s}=2\cdot \int_{a=0}^{q_{s}\cdot r_{s}}\rho \cdot \frac{da}{A_{i,s}\left(a\right)}=2\cdot \rho \cdot \int_{a=0}^{q_{s}\cdot r_{s}}\frac{da}{r_{s}^{2}\cdot \left(4-\pi \right)+\pi \cdot a^{2}}\\ =\frac{2\cdot \rho }{\sqrt{\left(4-\pi \right)\cdot \pi }\cdot r_{s}}\cdot {\left.arctan\left(\sqrt{\frac{\pi }{4-\pi }}\cdot \frac{a}{r_{s}}\right)\right|}_{a=0}^{q_{s}\cdot r_{s}}\\ =\frac{2\cdot \rho }{\sqrt{\left(4-\pi \right)\cdot \pi }\cdot r_{s}}\cdot arctan\left(\sqrt{\frac{\pi }{4-\pi }}\cdot q_{s}\right)=∶\frac{2\cdot \rho }{r_{s}}\cdot B_{i,s}(q_{s}). \end{array}$$

For the grain boundaries, it is assumed that the contact point between two intermediate grains is as shown in Figure 8c, i.e.,

(18) $$A_{i,gb,s}=A_{i,s}\left(q_{s}\cdot r_{s}\right)=r_{s}^{2}\cdot \left(4-\pi \cdot \left(1-q_{s}^{2}\right)\right).$$

Thus

(19) $$R_{i,gb,s}=f\cdot \rho \cdot \frac{l_{i,s}^{gb}}{r_{s}^{2}\cdot \left(4-\pi \cdot \left(1-q_{s}^{2}\right)\right)}.$$

The number of grains and grain boundaries is the same as for the grains. Thus, the electrical resistance of one row of intermediate grains $R_{i}^{s}$ is given by:

(20) $$R_{i}^{s}=N_{g,s}\cdot {(R}_{i,g,s}+R_{i,gb,s})={\left[\frac{l_{tips}}{{2\cdot q}_{s}\cdot r_{s}}\right]}_{int}\cdot \frac{\rho }{r_{s}}\cdot \left(2\cdot B_{i,s}(q_{s})+\frac{f\cdot l_{s}^{gb}}{r_{s}\cdot \left(4-\pi \cdot \left(1-q_{s}^{2}\right)\right)}\right).$$

### 3.2. Resistance Measurements of a Surface Layer/Coating on Top of Bulk Material

In the following, the factor *P*_m,n_ (distance-dependent resistance increase) and the electrical resistance of grain rows for three different grain shapes are derived separately for the surface layer/coating and the bulk material. Using these individual equations, the electrical resistance of combined layers can be calculated. For this, all rows have to be connected in parallel (i.e., sum the reciprocals of the electrical resistances of all rows in the surface layer/coating and the bulk material) in order to fit the modeled resistance to the measured one. The limits of *m*_L_, *m*_L,i_, *n*_L_, and *n*_L,i_ are given by the thickness and width of the surface layer/coating. The limits of *m*, *m*_i_, *n*, and *n*_i_ are determined by the thickness and width of the bulk material.

#### 3.2.1. Surface Layer/Coating with Cylindrically Shaped Grains—Resistance Measurement Parallel to the CA

Based on Figure 2 and Equation (2), $P_{L,CA,m_{L},n_{L}}$ is given by:

(21) $$P_{L,CA,m_{L},n_{L}}={\left(\frac{x_{L,CA}}{C_{L}}\right)}^{\gamma_{L}}={\left(\frac{2\cdot r_{L,c}}{C_{L}}\cdot \sqrt{{\left(m_{L}+0.5\right)}^{2}+n_{L}^{2}}\right)}^{\gamma_{L}}.$$

Using Equation (5), the total resistance of each row of grains can be calculated using

(22) $$R_{L,m_{L},n_{L}}^{CA}={\left(\frac{2\cdot r_{L,c}}{C_{L}}\cdot \sqrt{{\left(m_{L}+0.5\right)}^{2}+n_{L}^{2}}\right)}^{\gamma_{L}}\cdot \frac{\rho_{L}}{\pi \cdot r_{L,c}^{2}}\cdot \left(l_{tips}-{\left[\frac{l_{tips}}{h_{L}}\right]}_{int}\cdot l_{L,CA}^{gb}\cdot \left(1-f_{L}\right)\right).$$

For the intermediate volumes, $P_{L,i,CA,m_{L,i},n_{L,i}}$ becomes:

(23) $$P_{L,i,CA,m_{L,i},n_{L,i}}={\left(\frac{x_{L,i,CA}}{C_{L}}\right)}^{\gamma_{L}}={\left(\frac{2\cdot r_{L,c}}{C_{L}}\cdot \sqrt{m_{L,i}^{2}+{\left(n_{L,i}-0.5\right)}^{2}}\right)}^{\gamma_{L}}.$$

Combined with Equation (7), the total resistance of one row of intermediate volumes is given by (the area is halved for *m*_L,i_ = 0 and *m*_L,i_ = *m*_L,i,max_):

(24) $$\begin{array}{c} R_{L,i,m_{L,i},n_{L,i}}^{CA}={\left(\frac{2\cdot r_{L,c}}{C_{L}}\cdot \sqrt{m_{L,i}^{2}+{\left(n_{L,i}-0.5\right)}^{2}}\right)}^{\gamma_{L}}\cdot \frac{\rho_{L}}{\left(4-\pi \right)\cdot r_{L,c}^{2}}\\ \cdot \left(l_{tips}-{\left[\frac{l_{tips}}{h_{L}}\right]}_{int}\cdot l_{L,CA}^{gb}\cdot \left(1-f_{L}\right)\right)\;\left(\mathrm{for}\;m_{L,i}\in (0,m_{L,i,max})\right)\;\mathrm{and} \end{array}$$

(25) $$\begin{array}{c} R_{L,i,0,n_{L,i}}^{CA}={\left(\frac{2\cdot r_{L,c}}{C_{L}}\cdot \sqrt{m_{L,i}^{2}+{\left(n_{L,i}-0.5\right)}^{2}}\right)}^{\gamma_{L}}\cdot \frac{2\cdot \rho_{L}}{\left(4-\pi \right)\cdot r_{L,c}^{2}}\\ \cdot \left(l_{tips}-{\left[\frac{l_{tips}}{h_{L}}\right]}_{int}\cdot l_{L,CA}^{gb}\cdot \left(1-f_{L}\right)\right)\;\left(\mathrm{for}\;m_{L,i}\in \{0,m_{L,i,max}\}\right). \end{array}$$

#### 3.2.2. Surface Layer/Coating with Cylindrically Shaped Grains—Resistance Measurement Perpendicular to the CA

Based on Figure 2 and Equation (2), $P_{L,perp,m_{L},n_{L}}$ can be determined:

(26) $$P_{L,perp,m_{L},n_{L}}={\left(\frac{x_{L,perp}}{C_{L}}\right)}^{\gamma_{L}}={\left(\frac{1}{C_{L}}\cdot \sqrt{{\left(\left(m_{L}+0.5\right)\cdot 2\cdot r_{L,c}\right)}^{2}+{\left(n_{L}\cdot h_{L}\right)}^{2}}\right)}^{\gamma_{L}}.$$

Using Equation (12), the total resistance of each row of grains can be calculated by:

(27) $$R_{L,m_{L},n_{L}}^{perp}={\left(\frac{1}{C_{L}}\cdot \sqrt{{\left(\left(m_{L}+0.5\right)\cdot 2\cdot r_{L,c}\right)}^{2}+{\left(n_{L}\cdot h_{L}\right)}^{2}}\right)}^{\gamma_{L}}\cdot {\left[\frac{l_{tips}}{{2\cdot q}_{L,c}\cdot r_{L,c}}\right]}_{int}\cdot \frac{\rho_{L}}{h_{L}}\;\cdot \left(B_{L,c}\left(q_{L,c}\right)+\frac{f_{L}\cdot l_{L,perp}^{gb}}{2\cdot r_{L,c}\cdot \sqrt{1-{q_{L,c}}^{2}}}\right).$$

#### 3.2.3. Surface Layer/Coating with Spherically Shaped Grains

Based on Figure 2 and Equation (2), $P_{L,s,m_{L},n_{L}}$ is given by:

(28) $$P_{L,s,m_{L},n_{L}}={\left(\frac{x_{L,s}}{C_{L}}\right)}^{\gamma_{L}}={\left(\frac{2\cdot r_{L,s}}{C_{L}}\cdot \sqrt{{\left(m_{L}+0.5\right)}^{2}+n_{L}^{2}}\right)}^{\gamma_{L}}.$$

Utilizing Equation (16), the total resistance of one row of grains is:

(29) $$R_{L,m_{L},n_{L}}^{s}={\left(\frac{2\cdot r_{L,s}}{C_{L}}\cdot \sqrt{{\left(m_{L}+0.5\right)}^{2}+n_{L}^{2}}\right)}^{\gamma_{L}}\cdot {\left[\frac{l_{tips}}{{2\cdot q}_{L,s}\cdot r_{L,s}}\right]}_{int}\cdot \frac{\rho_{L}}{\pi \cdot r_{L,s}}\;\cdot \left(2\cdot B_{L,s}(q_{L,s})+\frac{{f_{L}\cdot l}_{L,s}^{gb}}{r_{L,s}\cdot \left(1-q_{L,s}^{2}\right)}\right).$$

For the intermediate volumes, $P_{L,i,s,m_{L,i},n_{L,i}}$ can be derived from Figure 2 and Equation (2):

(30) $$P_{L,i,s,m_{L,i},n_{L,i}}={\left(\frac{x_{L,i,s}}{C_{L}}\right)}^{\gamma_{L}}={\left(\frac{2\cdot r_{L,s}}{C_{L}}\cdot \sqrt{m_{L,i}^{2}+{\left(n_{L,i}-0.5\right)}^{2}}\right)}^{\gamma_{L}}.$$

Thus with Equation (20), the total resistance of each row of intermediate volumes is given by (the area is halved for *m*_L,i_ = 0 and *m*_L,i_ = *m*_L,i,max_):

(31) $$R_{L,i,m_{L,i},n_{L,i}}^{s}={\left(\frac{2\cdot r_{L,s}}{C_{L}}\cdot \sqrt{m_{L,i}^{2}+{\left(n_{L,i}-0.5\right)}^{2}}\right)}^{\gamma_{L}}\cdot {\left[\frac{l_{tips}}{{2\cdot q}_{L,s}\cdot r_{L,s}}\right]}_{int}\cdot \frac{\rho_{L}}{r_{L,s}}\;\cdot \left(2\cdot B_{L,i,s}(q_{L,s})+\frac{f_{L}\cdot l_{L,s}^{gb}}{r_{L,s}\cdot \left(4-\pi \cdot \left(1-q_{L,s}^{2}\right)\right)}\right)\;\left(\mathrm{for}\;m_{L,i}\in (0,m_{L,i,max})\right)\;\mathrm{and}$$

(32) $$R_{L,i,m_{L,i},n_{L,i}}^{s}={\left(\frac{2\cdot r_{L,s}}{C_{L}}\cdot \sqrt{m_{L,i}^{2}+{\left(n_{L,i}-0.5\right)}^{2}}\right)}^{\gamma_{L}}\cdot {\left[\frac{l_{tips}}{{2\cdot q}_{L,s}\cdot r_{L,s}}\right]}_{int}\cdot \rho_{L}\;\cdot \left(4\cdot B_{L,i,s}(q_{L,s})+\frac{f_{L}\cdot l_{L,s}^{gb}}{r_{L,s}\cdot \left(4-\pi \cdot \left(1-q_{L,s}^{2}\right)\right)}\right)\;\left(\mathrm{for}\;m_{L,i}\in \{0,m_{L,i,max}\}\right)$$

#### 3.2.4. Bulk Material with Cylindrically Shaped Grains—Resistance Measurement Parallel to the CA

In the model, the electrical resistance of the bulk material is impacted by the thickness *d*_L_ of the surface layer/coating. Using Figure 2, the factor *P* can be calculated for cylindrically shaped grains parallel to the CA under the assumption that *d*_L_ consists of an integer number of grains:

(33) $$P_{CA,m,n}={\left(\frac{x_{CA}}{C}\right)}^{\gamma }={\left(\frac{2\cdot r_{c}}{C}\cdot \sqrt{{\left({\left[\frac{d_{L}}{2\cdot r_{L,c}}\right]}_{int}\cdot \frac{r_{L,c}}{r_{c}}+m+0.5\right)}^{2}+n^{2}}\right)}^{\gamma }.$$

Combined with Equation (5), the total resistance of one row of grains is:

(34) $$R_{m,n}^{CA}={\left(\frac{2\cdot r_{c}}{C}\cdot \sqrt{{\left({\left[\frac{d_{L}}{2\cdot r_{L,c}}\right]}_{int}\cdot \frac{r_{L,c}}{r_{c}}+m+0.5\right)}^{2}+n^{2}}\right)}^{\gamma }\cdot \frac{\rho }{\pi \cdot r_{c}^{2}}\;\cdot \left(l_{tips}-{\left[\frac{l_{tips}}{h}\right]}_{int}\cdot l_{CA}^{gb}\cdot \left(1-f\right)\right).$$

Similarly for the intermediate volumes, $P_{i,CA,m_{i},n_{i}}$ is given by:

(35) $$P_{i,CA,m_{i},n_{i}}={\left(\frac{x_{i,CA}}{C}\right)}^{\gamma }={\left(\frac{2\cdot r_{c}}{C}\cdot \sqrt{{\left({\left[\frac{d_{L}}{2\cdot r_{L,c}}\right]}_{int}\cdot \frac{r_{L,c}}{r_{c}}+m_{i}\right)}^{2}+{\left(n_{i}-0.5\right)}^{2}}\right)}^{\gamma }.$$

Thus, the total resistance of each row of intermediate volumes can be calculated using Equation (7) (the area is halved for *m*_i_ = 0 and *m*_i_ = *m*_i,max_):

(36) $$R_{i,m_{i},n_{i}}^{CA}={\left(\frac{2\cdot r_{c}}{C}\cdot \sqrt{{\left({\left[\frac{d_{L}}{2\cdot r_{L,c}}\right]}_{int}\cdot \frac{r_{L,c}}{r_{c}}+m_{i}\right)}^{2}+{\left(n_{i}-0.5\right)}^{2}}\right)}^{\gamma }\cdot \frac{\rho }{\left(4-\pi \right)\cdot r_{c}^{2}}\;\cdot \left(l_{tips}-{\left[\frac{l_{tips}}{h}\right]}_{int}\cdot l_{CA}^{gb}\cdot \left(1-f\right)\right)\;\left(\mathrm{for}\;m_{i}\in (0,m_{i,max})\right)\;\mathrm{and}$$

(37) $$R_{i,m_{i},n_{i}}^{CA}={\left(\frac{2\cdot r_{c}}{C}\cdot \sqrt{{\left({\left[\frac{d_{L}}{2\cdot r_{L,c}}\right]}_{int}\cdot \frac{r_{L,c}}{r_{c}}\right)}^{2}+{\left(n_{i}-0.5\right)}^{2}}\right)}^{\gamma }\cdot \frac{2\cdot \rho }{\left(4-\pi \right)\cdot r_{c}^{2}}\;\cdot \left(l_{tips}-{\left[\frac{l_{tips}}{h}\right]}_{int}\cdot l_{CA}^{gb}\cdot \left(1-f\right)\right)\;\left(\mathrm{for}\;m_{i}\in \{0,m_{i,max}\}\right)$$

#### 3.2.5. Bulk Material with Cylindrically Shaped Grains—Resistance Measurement Perpendicular to the CA

Based on Figure 2 and Equation (2), $P_{perp,m,n}$ for cylindrically shaped grain perpendicular to the CA in the bulk material is given by:

(38) $$P_{perp,m,n}={\left(\frac{x_{perp}}{C}\right)}^{\gamma }={\left(\frac{1}{C}\cdot \sqrt{{\left({\left[\frac{d_{L}}{2\cdot r_{L,c}}\right]}_{int}\cdot 2\cdot r_{L,c}+\left(m+0.5\right)\cdot 2\cdot r_{c}\right)}^{2}+{\left(n\cdot h\right)}^{2}}\right)}^{\gamma }.$$

Then, the total resistance of one row of grains can be calculated by using Equation (12):

(39) $$R_{m,n}^{perp}={\left(\frac{1}{C}\cdot \sqrt{{\left({\left[\frac{d_{L}}{2\cdot r_{L,c}}\right]}_{int}\cdot 2\cdot r_{L,c}+\left(m+0.5\right)\cdot 2\cdot r_{c}\right)}^{2}+{\left(n\cdot h\right)}^{2}}\right)}^{\gamma }\;\cdot {\left[\frac{l_{tips}}{{2\cdot q}_{c}\cdot r_{c}}\right]}_{int}\cdot \frac{\rho }{h}\cdot \left(B_{c}\left(q_{c}\right)+\frac{f\cdot l_{perp}^{gb}}{2\cdot r_{c}\cdot \sqrt{1-{q_{c}}^{2}}}\right).$$

#### 3.2.6. Bulk Material with Spherically Shaped Grains

For spherically shaped grains in the bulk material using Figure 2 and Equation (2), $P_{s,m,n}$ is given by:

(40) $$P_{s,m,n}={\left(\frac{x_{s}}{C}\right)}^{\gamma }={\left(\frac{1}{C}\cdot \sqrt{{\left({\left[\frac{d_{L}}{2\cdot r_{L,s}}\right]}_{int}\cdot 2\cdot r_{L,s}+\left(m+0.5\right)\cdot 2\cdot r_{s}\right)}^{2}+{\left(2\cdot n\cdot r_{s}\right)}^{2}}\right)}^{\gamma }.$$

Applying Equation (16), the total resistance of one row of grains is:

(41) $$R_{m,n}^{s}={\left(\frac{1}{C}\cdot \sqrt{{\left({\left[\frac{d_{L}}{2\cdot r_{L,s}}\right]}_{int}\cdot 2\cdot r_{L,s}+\left(m+0.5\right)\cdot 2\cdot r_{s}\right)}^{2}+{\left(2\cdot n\cdot r_{s}\right)}^{2}}\right)}^{\gamma }\;\cdot {\left[\frac{l_{tips}}{{2\cdot q}_{s}\cdot r_{s}}\right]}_{int}\cdot \frac{\rho }{\pi \cdot r_{s}}\cdot \left(2\cdot B_{s}(q_{s})+\frac{{f\cdot l}_{s}^{gb}}{r_{s}\cdot \left(1-q_{s}^{2}\right)}\right).$$

The factor $P_{i,s,m_{i},n_{i}}$ (intermediate volumes) can be determined using Figure 2 and Equation (2):

(42) $$P_{i,s,m_{i},n_{i}}={\left(\frac{x_{i,s}}{C}\right)}^{\gamma }={\left(\frac{1}{C}\cdot \sqrt{{\left({\left[\frac{d_{L}}{2\cdot r_{L,s}}\right]}_{int}\cdot 2\cdot r_{L,s}+2\cdot m_{i}\cdot r_{s}\right)}^{2}+{\left(\left(n_{i}-0.5\right)\cdot 2\cdot r_{s}\right)}^{2}}\right)}^{\gamma }.$$

Using Equation (20), the total resistance of each row of intermediate volumes is given by (the area is halved for *m*_i_ = 0 and *m*_i_ = *m*_i,max_):

(43) $$R_{i,m_{i},n_{i}}^{s}={\left(\frac{1}{C}\cdot \sqrt{{\left({\left[\frac{d_{L}}{2\cdot r_{L,s}}\right]}_{int}\cdot 2\cdot r_{L,s}+2\cdot m_{i}\cdot r_{s}\right)}^{2}+{\left(\left(n_{i}-0.5\right)\cdot 2\cdot r_{s}\right)}^{2}}\right)}^{\gamma }\cdot {\left[\frac{l_{tips}}{{2\cdot q}_{s}\cdot r_{s}}\right]}_{int}\cdot \frac{\rho }{r_{s}}\;\cdot \left(2\cdot B_{i,s}(q_{s})+\frac{f\cdot l_{s}^{gb}}{r_{s}\cdot \left(4-\pi \cdot \left(1-q_{s}^{2}\right)\right)}\right)\;\left(\mathrm{for}\;m_{i}\in (0,m_{i,max})\right)\;\mathrm{and}$$

(44) $$R_{i,m_{i},n_{i}}^{s}={\left(\frac{1}{C}\cdot \sqrt{{\left({\left[\frac{d_{L}}{2\cdot r_{L,s}}\right]}_{int}\cdot 2\cdot r_{L,s}+2\cdot m_{i}\cdot r_{s}\right)}^{2}+{\left(\left(n_{i}-0.5\right)\cdot 2\cdot r_{s}\right)}^{2}}\right)}^{\gamma }\cdot {\left[\frac{l_{tips}}{{2\cdot q}_{s}\cdot r_{s}}\right]}_{int}\cdot \frac{\rho }{r_{s}}\;\cdot \left(4\cdot B_{i,s}(q_{s})+\frac{f\cdot l_{s}^{gb}}{r_{s}\cdot \left(4-\pi \cdot \left(1-q_{s}^{2}\right)\right)}\right)\;\left(\mathrm{for}\;m_{i}\in \{0,m_{i,max}\}\right)$$

## 4. Discussion

The model equations include a variety of parameters that have to be determined. They are summarized in Table 1 along with a possible method of determination.

Resistance offsets (*R*_off_, *R*_off, L_) can be added to the equations to account for further unknown/unconsidered factors, inaccuracies caused by assumptions made during modeling (e.g., parameter ranges/distributions, which are modeled with one single value), or experimental factors, such as the electrical resistance of cables or within the four-point probe.

Due to the complexity of the models, the calculations require an experimental calibration of the included material-specific parameters for calibration samples with known grain shape and grain size parameters (i.e., *q*, *h*, and *r*). Depending on the materials and grain shapes in the surface layer/coating and the bulk, up to six individual parameters are required (i.e., *C*, *C*_L_, *γ*, *γ*_L_, *f*, and *f*_L_*)*. However, they are expected to be material-specific parameters. Thus, they can be determined using homogeneous samples and stored in a database to be re-used when the same material is investigated later on. Therefore, each material requires a minimum of four to five calibration samples to fit the model parameters. Homogeneous calibration samples can be produced by, e.g., heat treatments or rolling.

The models can be simplified in order to reduce calibration efforts and microstructural investigations:

- γ can be considered constant, i.e., identical for all materials or identical for the surface layer/coating and bulk material.
- Grain boundaries can be combined with the above-mentioned resistance offsets *R*_off_ and *R*_off, L_. Then, the grain-boundary-related terms in the equations can be omitted, which leads to the elimination of the parameters *f* and *l*_gb_ from the equations. In addition, *q*_c_ = 1 can be used in the case of cylindrically shaped grains measured perpendicular to the CA when grain boundaries are not explicitly included.

After the calibration is complete, real samples can be measured. Under the assumption that *q* is known, each model includes one or two geometry-related parameters both for the surface layer/coating and the bulk material, which describe the grains. Thus, one individual resistance measurement does not provide enough information to determine the grain size parameters from the measured electrical resistance. Therefore, the electrical resistance of the bulk material could either be measured separately (i.e., without surface layer/coating and model equation with *d*_L_ = 0), or its grain size parameters could be determined experimentally (e.g., microscopically). Thus, under the assumption that the bulk material is unaltered by the formation of the surface layer/coating, the radius of spherically shaped grains in the surface layer/coating can easily be calculated from the measured electrical resistance. For cylindrically shaped grains, however, there are two unknown parameters, which leads to four possible approaches. Approach (1) is suitable for most samples. However, if there are limitations, e.g., regarding the sample geometry, approaches (2)–(4) can be used:

1. The sample needs to be measured in two directions, i.e., parallel and perpendicular to the CA of the cylindrically shaped grains.
2. The sample can be measured in one direction if one of the geometry-related parameters is already known, e.g., from microstructural investigations.
3. Two different samples can be measured in one direction if one of the geometry-related parameters is constant in both samples.
4. Two samples with an identical grain size and shape but different *d*_L_ can be measured in one direction, e.g., different coating thicknesses.

The models are subject to the following limitations when considering real samples:

- Grain size and shape
  ○ There is a distribution of grain sizes and—possibly—grain shapes, which cannot be described by the models. Only mean sizes are considered.
  ○ Typically, the grain shape is irregular. The models consider mean shapes only.
  ○ The grain size and shape can gradually change near the interface between the surface layer/coating and bulk, which cannot be described by the models.
  ○ Typically, the grains are not perfectly aligned in rows, which causes deviations between the modeled and the measured electrical resistances.
  ○ The assumption of a strict electrical separation of the rows of grains is likely not entirely fulfilled.
  ○ The maximum grain size is limited by the measurement range and resolution of the four-point probe device. With increasing grain size, the measured resistance decreases and the resistance change diminishes, which reduces the accuracy when matching the experiment and model. The maximum grain size is expected to be about 200 µm for materials with a low resistivity.
  ○ For grain sizes above 1 µm, the resistivity is expected to be constant. As this is a crucial requirement when comparing different samples, the grain sizes should not be significantly smaller than 1 µm.
- Microstructural features
  ○ The chemical composition and thus the resistivity *ρ* can gradually change near the interface, e.g., for coatings after a heat treatment, which cannot be described by the models.
  ○ The porosity of the surface layer/coating or the bulk material is not explicitly considerer in the model. Porosity can cause deviations in the form of seemingly lower grain sizes. An adjusted resistivity can be used to account for porosity.
  ○ Second phases are not included in the models. Precipitates can be partially accounted for by an adjusted resistivity. However, larger phase fractions of a secondary phase would add an additional set of parameters, which cannot be determined accurately with adequate experimental effort.
  ○ Surface roughness as well as rough interfaces between the surface layer/coating and bulk material cannot be described by the models. Flat surfaces and interfaces are assumed.
- Material: Although no explicit assumptions are made that limit the materials, the following restrictions have to be considered:
  ○ In semiconductors, microstructural features (such as residual stresses in the material or as a consequence of a coating process) or doping can change the resistivity of the material significantly. Thus, the model calibration cannot be performed easily, and the models should only be applied to metals.
  ○ If the resistivities of the surface layer/coating and substrate differ significantly, then the highly resistive material will have a very low impact and might not be derived from the models within the experimental accuracy.
- Layer thickness:
  ○ For very thick layers with high resistivity, the substrate only has a small impact on the measured resistance value. Thus, within the experimental limits, the grain size of the substrate cannot be calculated accurately.
  ○ The low impact of surface layers/coatings with only a small thickness or with a limited difference from the underlying bulk material on the measured resistance will cause inaccurate experimental results, which prevent accurate calculations. Additionally, thin layers might not cover the surface completely or might be penetrated by the four-point probe tips, which would make them unsuitable for measurement.
  ○ Local variations in the thickness *d*_L_ cannot be described by the models.

## 5. Conclusions

Based on simple geometric considerations, the previous models [21] were extended to now include:

- grain boundaries;
- intermediate volumes, i.e., no hollow volumes in the material;
- double-layered systems with different grain shapes and sizes in each area.

As almost all machining processes cause the formation of surface layers, the extended models can be applied to real processes and parts. Metallic coatings, which are of great importance, e.g., for corrosion and wear protection of metallic parts, can also be described. Even systematic deviations caused by the used model assumptions can be neglected if the models are applied for the non-destructive quality control of parts to ensure sufficiently low variations in grain sizes and thus, e.g., high fatigue strengths. In this case, only changes during production, which can be caused by tool wear, would be relevant. 

Despite the mentioned limitations, the models provide the possibility to calculate grain sizes of systems which consist of a surface layer/coating and the underlying bulk material for metallic materials the first time. Table 2 summarizes the relevant equations, which can be used for the calculations.

Using the modeling approaches, which are described here, models for further grain shapes can be derived. Relevant examples are ellipsoids, upright cylinders, and cylinders with semi-spheres attached to their base planes. In further studies, the models will be tested on electrodeposited coatings.

## Figures and Tables

**Table 1:** Model parameters and a possible source of determination (*l*_tips_—distance between the inner tips of the four-point probe; *ρ*—(volume) resistivity of the material; *d*_L_—thickness of the surface layer/coating; *q*—flattening factor of the grains; *l*^gb^—thickness of one individual grain boundary; *C*—resistance-increasing factor; *γ*—resistance-increasing power; *f*—resistivity increase of grain boundaries).

Parameters	Source of Determination
*l*_tips_	measured from the used four-point probe
*ρ*	literature
grain shapes, *d*_L_, *q*, *l*^gb^	investigation of the microstructure or estimation
*C*, *γ*, *f*	fitting of calibration samples

**Table 2:** Overview of the relevant equations for different grain shapes.

	Parallel to CA	Perpendicular to CA	Spherical
surface layer/coating	(22), (24), (25)	(27)	(29), (31), (32)
bulk material	(34), (36), (37)	(39)	(41), (43), (44)

## Data Availability

Data sharing not applicable.
